# Overview of Mendelian Susceptibility to Mycobacterial Diseases (MSMD)

**DOI:** 10.7759/cureus.85872

**Published:** 2025-06-12

**Authors:** Kausalya Raghuraman, Rajeswarie S, Purnima Rajkhowa, Jaya S Kaushik

**Affiliations:** 1 Microbiology, All India Institute of Medical Sciences, Guwahati, Guwahati, IND; 2 Pediatrics, All India Institute of Medical Sciences, Guwahati, Guwahati, IND

**Keywords:** bcg vaccine, mendelian, mendelian susceptibility to mycobacterial diseases, microbial genetics, mycobacterial disease, tuberculosis

## Abstract

Mendelian susceptibility to mycobacterial diseases (MSMD) refers to a group of genetic conditions predisposing an individual to environmental mycobacteria and other intracellular pathogens, leading to disseminated infection. Nine MSMD genes have been identified, which include seven autosomal (IFNGR1, IFNGR2, STAT1, IL12B, IL12RB1, ISG15, and IRF8) and two X-linked (NEMO and CYBB) genes. MSMD patients present with disseminated BCGosis or with symptoms of non-tuberculous *Mycobacterium* (NTM). Host defense mechanisms, such as the interferon (IFN) gamma and IL-12 pathways, which activate macrophages, play a crucial role in combating the *Mycobacterium* species. Treatment with interferon gamma and hematopoietic stem cell transplantation holds promise.

## Introduction and background

Mendelian susceptibility to mycobacterial diseases (MSMD) is a group of genetic disorders that predispose individuals to clinical disease caused by the bacillus Calmette-Guérin (BCG) vaccine and environmental mycobacteria, with no overt abnormality in immunological and hematological parameters, among the healthy population [1]. Immunocompromised individuals, such as HIV infected individuals, with severe or disseminated infection due to nontuberculous mycobacteria, produce high rates of mortality and morbidity.

MSMD is a form of primary immunodeficiency and is not typically found in individuals without underlying genetic susceptibility [2]. MSMD, a relatively rare genetic disorder, affects one in 100,000 individuals [3]. Disseminated BCG infection is also seen in patients with HIV, severe combined immunodeficiency, and chronic granulomatous disease. These immunocompromised conditions are more common and are amenable to treatment. Hence, these conditions must be ruled out before MSMD is considered a possible diagnosis and etiology for disseminated BCG infection [4]. Nine MSMD genes have been identified to date, including seven autosomal (IFNGR1, IFNGR2, STAT1, IL12B, IL12RB1, ISG15, and IRF8) and two X-linked (NEMO and CYBB) genes [1].

Primary tuberculosis usually presents with extrapulmonary manifestations in children due to the bacteria's dissemination into the bloodstream [5]. MSMD disease usually manifests in childhood, and they are also prone to more virulent mycobacterial tuberculosis. The disease spectrum of MSMD includes not only tuberculosis but also nontyphoidal salmonellosis. Some rare cases of listeriosis, nocardiosis, fungi (histoplasmosis), parasites (leishmania, toxoplasma), and viruses (Cytomegalovirus, human herpes virus 8) have also been reported [1].

The genetic etiology of MSMD was discovered only at the end of the 20th century [6]. Since then, 18 different disorders have been identified based on the inheritance pattern, complete or partial defect in the gene, expression of the mutant allele, and the function affected [1]. Complete IFNGR1 and IFNGR2 deficiency are associated with severe phenotypes of MSMD [7]. This review deals with the pathogenesis, clinical features, diagnosis, and treatment of Mendelian susceptibility to mycobacterial infections.

Search strategy

The articles in English on "Mendelian susceptibility to mycobacterial disease" were collected from PubMed and Google Scholar search engines from 1990 to 2025. The articles were mainly published as case reports and case reviews. All the cases that were genetically identified to have MSMD were included in the study.

## Review

Pathogenesis

The cohesion of mononuclear phagocytic cells and T lymphocytes is responsible for killing the mycobacterial infection. The T helper 1 cells produce interferon (IFN) γ, which is the principal eliminator of *Mycobacterium*. Either insufficient production of IFN gamma or inadequate response to it predisposes to MSMD [8].

**Figure 1:**
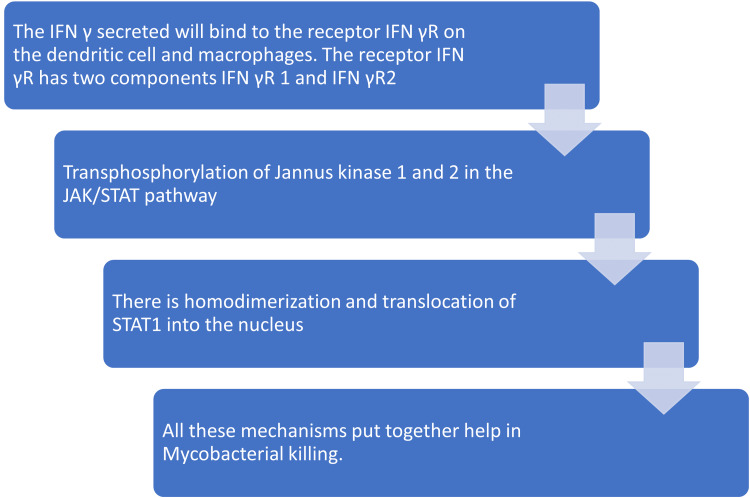
Interferon (IFN) pathway against Mycobacterium infection.

The host defense mechanism against mycobacterial infection is as follows [9-12]: antigen-presenting cells (dendritic cells and macrophages) recognize mycobacteria and secrete IL-12 and IL-23. IL-12, in turn, stimulates T helper (TH) cells and natural killer (NK) cells through receptors. TH cells are transformed into TH1, which secretes IFN-γ, IL-17, and tumor necrosis factor-alpha (TNF-α). Two different pathways process IL-12 and IFN γ, one is the T cell-dependent pathway, and the other is by co-stimulation of receptors of the IL-1 receptor family, such as IL-18R (Figure 1).

Clinical features

MSMD patients present with disseminated BCGosis or with symptoms of non-tuberculous *Mycobacterium* (NTM). The nonspecific symptoms of NTM include fever, weight loss, lymphadenopathy, gastrointestinal symptoms, and skin lesions [13]. Severity of the disease depends on two factors: the gene involved and whether it is a partial or complete deficiency [14]. Clinical manifestations may differ in mild to severe infections. Mild infections can be late in onset and may manifest as isolated lymphadenopathy, local inflammatory reaction at the BCG site, or low-grade constitutional symptoms. In contrast, severe infection can manifest early in infancy with manifestations like disseminated multi-site infection, including osteomyelitis.

The various genes involved have been explained briefly below.

IFNGR1 deficiency

IFNGR1 encodes for the ligand-binding chain of interferon γ receptor [2]. A 22 kb IFNGR1 gene is on the 6q23.3 region [7]. The autosomal dominant and recessive mutations are known to produce this MSMD (Table 1) [15-20]. These patients with complete IFNGR deficiency fail to eradicate the infection due to antibiotic failure, and there is no response to interferon-gamma therapy [2].

**Table 1:** Demographic and genetic profile of patients affected with IFNGR1 deficiency. ATT: antitubercular treatment; IFN: interferon; cDNA: complementary DNA; TB: tuberculosis; BCG: bacillus Calmette-Guérin.

Type of mutation	Place, year	Age, gender (M: male; F: female) of the patient	Consanguinity	Main symptom	Infection	Others
Tsp 451 [6]	Malta, 1996	1 year, M	Yes	Fever, anorexia, night sweats, splenomegaly	M. chelonae	Erythromycin, prednisolone; succumbed to the illness
		3 years, M	-	Fever, night sweats, lymphadenopathy, splenomegaly	M. avium intracellulare	ATT + prednisolone + gamma interferon; succumbed to the illness
		15 months, M	-	Fever, weight loss, hepatosplenomegaly	M. fortuitum	Anti-atypical mycobacterial drugs and interferon
		2 years and 9 months, F	-	Fever, anorexia, weight loss	M. avium intracellulare	ATT + antimicrobials; succumbed to the illness
Mutation [15]	Norway, 1998	5 years, M	Yes	Weight loss, anemia, generalized lymphadenopathy	M. avium complex	Antitubercular treatment; succumbed to the illness
		2 years, M	-	Weight loss fever, hepatosplenomegaly	-	Antitubercular treatment and interferon gamma
Mutation in exon 3 [16]	Greece, 1999	7 years, F	No	Lymphadenopathy, hepatosplenomegaly	BCG, M. kansasii, M. avium intracellulare	Isoniazid, ethambutol, streptomycin, and rifampicin, followed by bone marrow transplant
Homozygous novel null mutation 453del T [2]	Greece, 2006	2 years, M	Remote consanguinity	Generalized lymphadenitis and rash	M. fortuitum, M. peregrinum	Amikacin, ciprofloxacin, clarithromycin. Poor prognosis
Homozygous 523 del T mutation [17]	Italy, 2010	2 years and 10 months, M	No	Fever, pain in the right leg and left hand, gait problem, abdominal pain	M. scrofulaceum	Clarithromycin, streptomycin, rifabutin, ethambutol
Homozygous mutation in exon 3 (c.182 A>G) [18]	Mexico, 2013	7 years, M	Yes	Lymphadenopathy	M. gordonae	Rifampicin, pyrazinamide, and clindamycin for 18 months
Insertion of 21bp in cDNA [18]		15 years, F	No	Urethritis, oral ulcer, cerebral TB, bone TB	BCG	Succumbed to illness
Homozygous mutation in exon 7 [18]		4 years, M	No	Lymphadenopathy	BCG	Isoniazid, stem transplant
Homozygous mutation in exon 9 [18]		8 months, F	No	Lymphadenopathy, fever, hepatosplenomegaly	BCG	Rifampicin, pyrazinamide, isoniazid, IFN gamma
Homozygous mutation in exon 7 [18]		4 years, M	No	Syncope and paraparesis	BCG	Rifampicin, pyrazinamide, isoniazid
c.114_135del(p.E38fsX54) [19]	China, 2014	18 months, F	Yes	Right axillary lymph node followed by bone tuberculosis	Post BCG vaccination	Rifampicin, isoniazid, amoxicillin clavulanic acid
119.227nt homozygous deletion on chromosome 6q 23.3 and ending close to IL22RA2 gene [7]	Turkey, 2016	1 year, F	Yes	Cervical lymphadenopathy	M.fortuitum	Ciprofloxacin and cotrimoxazole
Biallelic mutation in IFNGR1 gene, a homozygous nucleotide substitution C > A, leading to P130H [20]	India, 2021	5 months, F	Yes	Fever, rash, left axillary lymphadenitis, hepatosplenomegaly	M. bovis	Antitubercular treatment; succumbed to the illness

Most of the IFNGR1 patients presented with varied symptoms and resulted in a fatal outcome despite therapy.

IFNGR2 deficiency

IFNGR2 chain is associated with the ligand binding of IFNGR1 to form a complete receptor for IFN γ. It is not as common as IFNGR1 deficiency and is an infrequent inherited MSMD [21]. The autosomal recessive (AR) complete IFNGR2 deficiency is the most severe type of IFNGR2 deficiency, with frequent infections and a high mortality rate [22]. Studies have shown a genetic defect in IFNGR2 deficiency (Table 2) [2-25].

**Table 2:** Gene mutation, demographics, clinical features, and treatment of patients with MSMD IFNGR2 deficiency. ATT: antitubercular treatment; IFN: interferon; MSMD: Mendelian susceptibility to mycobacterial diseases.

Type of mutation	Place, year	Age of the patient	Consanguinity	Main symptom	Infection	Others
Amino acid substitution in the extracellular region [22]	Portugal, 2000	At birth, female	-	Disseminated granuloma	BCGosis	ATT treatment
		16 years	-	Cellulitis and adenitis	M. abscessus	ATT + INF gamma
791delG [23]	Holland, 2004	15 months, female	-	Lymphadenopathy, hepatosplenomegaly, and osteomyelitis of the right femur, tibia, and mandible	M. abscessus	ATT + INF gamma + bone marrow transplant
		4 months male	-	Fever, anemia, rash, and coagulopathy	M. avium	ATT
949delTG [20]	Japan, 2010	I year and 3 months	-	Lung abscess, hepatosplenomegaly	M. avium, M. fortuitum, M. porcinum	Kanamycin, isoniazid, rifampicin
Exon 4, nonsense mutation C958insT [21]	Islamic Arab, 2014	2.5 years, male	Yes	Lymph node at the ileocaecal junction	M. simiae	Cycloserine, clarithromycin
	Palestine, 2014	5 months	Yes	Lymphadenopathy	M. bovis	ATT
		3 years	Yes	Lytic bone and liver lesions	M. fortuitum	ATT + macrolide
		5 years	Yes	Brain abscess	M. simiae	Succumbed to infection
R679G>A [24]	Turkey, 2012	5 months, male	Distantly related	Axillary enlargement with fistula, fever, fatigue, and multiple lymph nodes	BCGosis, Mycobacterium tuberculosis complex	Isoniazid and rifampicin for 5 months, ATT

In IFNGR2 patients, lymphadenopathy was the predominant sign, and most of the patients improved with antitubercular treatment

STAT1 deficiency

STAT1 is a transcription factor involved in cytokine-mediated signaling [26]. Compared with other gene disorders in STAT deficiency, it is due to impairment and not deficiency of the enzyme, which leads to MSMD [27]. The STAT1 phosphorylation occurs by two mechanisms: IFNγ and IFNGR1 will lead to activation of JAK1/JAK2 receptors; the second mechanism is that IFN α will lead to phosphorylation of STAT2, both pathways leading to phosphorylation of STAT1 [28]. The phosphorylated STAT1 combines with the gamma activation sequence, resulting in an antimycobacterial effect. When STAT1 and STAT2 interact with IRF9, they activate interferon-stimulated gene factor 3 (ISGF3). ISGF3, along with IFN α, is responsible for the antiviral effect. Hence, a partial defect leads to MSMD, and a complete defect leads to a severe, life-threatening condition (Table 3) [26-32].

**Table 3:** Demographic and genetic profile of patients affected with STAT deficiency. BCG: bacillus Calmette-Guérin.

Type of mutation	Place, year	Age of the patient	Consanguinity	Main symptom	Infection	Others
Exon 20 deletion [29]	Paris, 2003	Infants	No	Disseminated BCG infection	BCGosis	Died due to a viral infection
Splicing out of exon 8 [27]	Saudi Arabia, 2010	6 years, M	Yes	Axillary lymphadenitis	M. avium	Cycloserine, ethionamide, ethambutol, and moxifloxacin
		3 years, F		Axillary lymphadenitis	BCGosis	Died due to septic shock
Mutation in the N-terminal region [28]	Denmark, 2011	14 years, F	-	Fever, weight loss, and bone pain	M. avium	Clarithromycin, rifampicin, ethambutol
Splicing mutation in exon 3 [30]	Italy, 2011	3 years	Yes	Pulmonary infection, respiratory distress	M. kansasii	Antimycobacterial and bone marrow transplant
Missense mutation in the SH2 region [26]	Japan, 2012	6 years, M	No	Multifocal osteomyelitis	Mycobacterium	Antimycobacterial
	Saudi Arabia, 2012	5 months, F	Yes	Osteomyelitis	BCGosis	Isoniazid, rifampicin, ciprofloxacin
Mutation in the SH2 domain c1961T>A [31]	Bethesda, 2012	5 years, M	No	Recurrent pneumonia and cervical lymphadenitis	M. avium complex	Ethambutol, azithromycin, rifampin, ciprofloxacin, interferon gamma
Inherited [32]	Israel, 2013	17 years, M	-	Multifocal osteomyelitis	M. szulgai	Ethambutol, rifampicin, and azithromycin

In patients with STAT deficiency, lymphadenitis and osteomyelitis were the most common clinical features seen, and they responded to the treatment.

IL12B

IL-12 is a heterodimer cytokine with two subunits, p35 and p40. IL-12 encodes the p40 subunit, which is a shared component of IL-12 and IL-23. This subunit plays a crucial role in immune responses, particularly in activating T cells and natural killer (NK) cells [33]. IL-12 encodes for IL-12p40, a common link in the pathway of IL-12 and IL-23. These patients generally have a good prognosis. They are treated with antibiotics and IFN γ therapy [1]. IL-12B deficiency has been noticed in all age groups with natives from Pakistan, India, Saudi Arabia, Tunisia, and Iran. They have been diagnosed with BCG infection, non-tubercular *Mycobacterium* (*M. chelonae*), and *Mycobacterium tuberculosis* (MTB) diseases. The various mutations found are g482+82_856-854, 278del 8, 179G>A;pTrp60X, 909insA, 526-528del CT, and 697+5G>A, and the most common is 315insA [34,35].

IL12RB1

The IL12RB1 gene encodes for IL12RB1 receptor, which has a high affinity for IL-12 binding and signaling. The spectrum varies from early death in infancy to an asymptomatic course in adulthood [4,8,20,36-38]. It is the most common MSMD for severe tuberculosis (TB) (Table 4) [1]. The spectrum varies from early death in infancy to disseminated tuberculosis, and severe malnutrition to cervical lymphadenopathy, and remains asymptomatic in adulthood.

**Table 4:** Demographic and genetic profile of patients affected with IL-12RB1 deficiency. BCG: bacillus Calmette-Guérin; HRZE: isoniazid, rifampin, pyrazinamide, and ethambutol.

Type of mutation	Place, year	Age of the patient	Consanguinity	Main symptom	infection	Others
Mutation in exon 2 and exon 14 [37]	Belgium, 2008	9 years, female	No	Cervical lymphadenopathy	M. avium	Amikacin, rifabutin, clarithromycin
Mutation in exon 12 (c677T and A 1298 C) [38]	Turkey, 2014	16 years, female	No	Cough, hepatosplenomegaly, and sudden unconsciousness	Disseminated M. tuberculosis with venous thrombosis	Isoniazid, rifampicin, ethambutol, and pyrazinamide
Complete deletion of IL12RB1 [4]	Sri Lanka, 2015	4 months	No	Left axillary lymph node enlargement and severe bronchopneumonia	Disseminated BCG vaccination	Isoniazid, rifampicin, ethambutol, and streptomycin for 4 months, followed by isoniazid and rifampicin for 4 months
Homozygous mutation c.1791+2T>G [8]	Iran, 2016	8 years	No	Lymphadenopathy and bloody diarrhea	Disseminated BCG vaccination	-
Frame shift deletion c.1172delC [8]		2.5 years		Lymphadenopathy, fistula, and petechiae		
		2.5 years		Generalized lymphadenopathy		
c.962C > A responsible for AR complete IL-12Rb1deficiency [20]	India, 2021	5 months, female	Yes	Left axillary adenopathy, severe malnutrition	M. bovis	HRZE & ofloxacin; there was a relapse after a year
Homozygous mutation in IL12RB1 gene, c.962C > A [20]		1 year, female	Yes	Left axillary lymphadenopathy, multiple neck swellings	-	Antitubercular treatment with linezolid and amikacin

A survey was conducted on 121 patients from 30 countries, including Sri Lanka, Iran, Russia, Turkey, Tunisia, Argentina, China, Morocco, Cameroon, Cyprus, Spain, Germany, Belgium, and Poland, to name a few. The patients with MSMD were in all age groups. The common mutations seen were G569D, 1791+2T>G, 64+2T>G, 1623-1624del TT, W531X, 1386-01387del GT, 711 ins C, R173P, Y88X, and C186S,1990-1G>A. The patients mostly presented with disseminated BCGosis, with a few cases due to *M. avium* complex [36].

ISG15

Interferon-stimulated gene 15 is a very potent inducer of IFN γ production. ISG15 is released upon bacterial challenge by the neutrophils and myeloid cells [1,39]. AR deficiency in ISG15 patients is treated with humanized ISG15, which shows tremendous improvement [39]. Six patients from Iran, Turkey, and China aged between 14 and 17 years reported with BCGosis. The mutation observed was c.379G>T/379G.T on exon 2. It was also noted that patients with this type of MSMD had cerebral calcifications leading to epileptic seizures [40,41].

IRF8

Interferon regulatory factor 8 (IRF8), expressed on macrophages and dendritic cells, regulates granulocyte and macrophage differentiation. The IRF8 protein binds to IFN-stimulated response elements and regulates genes stimulated by IFNα/β. Three children were diagnosed with the K108E and T80 mutation of IRF8 and presented with recurrent attacks of BCGosis. The three children are unrelated and from nonconsanguineous Italian patients. They were managed with antitubercular drugs [1,42].

X-linked recessive MSMD

MSMD-related NEMO and CYBB mutations are hypomorphic and do not always present with classic features of ectodermal dysplasia or chronic granulomatous disease. The E315A and R319Q mutations of NEMO disrupt the formation of salt bridges usually formed between the two entities, which leads to impairment of CD40-dependent IL-12 production. Hence, there would be a low level of IFN γ and IL-12 production [1].

In 1994, the first NEMO-related MSMD was described in four male patients from the USA, who were found to have the E315A mutation. Later, two boys, from Germany and France, were found to have the R319Q mutation. The patients developed disseminated tuberculosis and *M. avium* infection and were treated with antibiotics and interferon gamma therapy [1,43].

CYBB/gp91phox mutation results in decreased nicotinamide adenine dinucleotide phosphate (NADPH) oxidase activity. Chronic granulomatous disease (CGD) is an important feature of the CYBB mutation. Around 25% of CGD patients develop tuberculosis infection. Two hemizygous mutations of this gene are known: Q231P and T178P. Seven patients from two unrelated families developed BCGosis without CGD features. It was found that they had a normal respiratory burst activity [39].

Few MSMD cases have been reported from India, contributing to the expanding global genotype-phenotype correlation. One case of extensively drug-resistant (XDR) tuberculosis in a two-year-old boy with tubercular meningitis has been reported from Mumbai. On stimulation with BCG and BCG along with IFN γ, there was an increased production of IL12p40, suggestive of IL-12Rβ1 deficiency. However, this could not be genetically proven as the child succumbed to the illness [44 ]. Another six-year-old boy born to nonconsanguineous Indian parents presented with multifocal osteomyelitis due to *Mycobacterium* avium complex. The child had localized BCG lymphadenitis at one month of age. On analysis, the child was found to have a heterozygous mutation of exon 6,818 del T of the IFNGR1 gene. The child was treated with rifampicin, ethambutol, ofloxacin, and clarithromycin, along with interferon gamma, and the child improved with treatment [45]. Another report of a three-year-old female presented with multiple neck swellings, pallor, eczematous skin rash, and hepatosplenomegaly, and was found to have histiocytosis. On whole genome sequencing, it was found to be homozygous c.201-2A>G splice variant in intron 2 of the -1 gene. The child was diagnosed with MSMD due to *M. fortuitum* and treated with meropenem, amikacin, cefixime, clofazimine, levofloxacin, and interferon therapy [46].

Laboratory diagnosis

Suspicion of MSMD

A child who develops repeated infection with an NTM or BCGosis when other immunocompromised conditions have been ruled out should raise suspicion of MSMD. The history of siblings having similar features adds to clinical suspicion. Various differential diagnoses for MSMD include HIV infection, severe combined immunodeficiency, chronic granulomatous diseases, lung disorders like primary ciliary dyskinesia, pulmonary alveolar proteinosis, and cystic fibrosis, autosomal dominant GATA 2 deficiencies, anhidrotic ectodermal dysplasias, and X-linked recessive CD40L deficiency.

Diagnosis of a Specific Non-tuberculous Mycobacterium

The organism identification up to species level becomes important mainly for treatment purposes, as different NTM have different lengths of treatment. Radiological imaging helps us identify any lung involvement. Granulomatous reaction in tissue sections would lead to mycobacterial infection.

Acid-fast stain and culture: Culture done on Lowenstein-Jensen medium and incubated at 37°C for up to eight weeks and examined for growth weekly is considered as gold standard, as it is important for drug susceptibility testing and genotypic identification. The Gene Xpert/RIF assay primarily detects *Mycobacterium tuberculosis* complex and rifampin resistance, but is not always useful for identifying NTM species. Genotypic identification is done by line probe hybridization, polymerase chain reaction, and DNA sequencing [47].

Diagnosis of Mutation

The first step is to diagnose whether any deficiency is present in the IFN gamma pathway [48]. The interferon levels can be measured using enzyme-linked immunosorbent assay (ELISA) [3] and the leukocyte stimulation test.

Leukocyte stimulation test: In this method, the peripheral blood mononuclear cells are stimulated with phytohemagglutinin. The supernatant is then assayed for cytokine levels in Bio-Plex assays [48]. Flow cytometry is a sensitive tool in diagnosing all primary immunodeficiency disorders. In MSMD patients, aberrant IFN γR1 expression on monocytes or deficient IL-12RB1 expression on activated T cells can be assessed [49]. Once we have identified that there is a defect, then complementary DNA (cDNA) analysis is performed [7]. Previously, Sanger sequencing and exon sequencing were done to detect the mutation. Whole genome sequencing, whole exome sequencing, and next-generation sequencing have paved the way for genome sequencing within a single day [39,50].

In cases presenting with a lymphadenopathy, BCGosis, or disseminated tuberculosis, with a history of consanguinity, demise of a sibling with similar presentation, or repeated pregnancy loss of the mother, MSMD may be suspected. Isolation of NTM or disseminated tuberculosis in a previously healthy child could also be a suspected case of MSMD. These patients will have to be ruled out for other common immunodeficiencies like severe combined immunodeficiency or HIV. ELISA may be used to determine the level of IFN gamma or IL-12. However, if there is a strong clinical suspicion, then targeted genetic sequencing or whole genome sequencing would be the most useful, as it would detect the genetic defect.

Treatment

MSMD treatment includes identifying the gene mutation, as the treatment depends on the mutated gene and identification of the nontuberculous infection or BCGosis [48]. Additional treatment options are cytokine and interferon therapy, which will benefit in IL12B and IL12B1, and STAT1 therapy [1]. NTM is treated with antitubercular antibiotics till the patient is clear of infection microscopically and radiologically. Mycobacterial avium complex is treated with macrolide, ethambutol, and rifamycin for 18-24 months. *M. kansasii* infections are treated with isoniazid, ethambutol, and rifamycin for 12 months [47]. Hematopoietic stem cell transplant is indicated in severe cases, especially in IFNGR1 and IFNGR2 MSMD cases [1]. Early recognition and gene-guided therapy, including IFN-γ or hematopoietic stem cell transplantation when indicated, can significantly improve outcomes in affected children [51].

## Conclusions

MSMD represents an emerging group of primary immunodeficiencies underlying susceptibilities to NTM. Host defense mechanisms like the IFN gamma and IL-12 pathways are important against the mycobacterium species. Hence, any mutation at these pathways will not curtail the infection. Few studies from India have been quoted regarding MSMD.
